# Chinese Version of the Assessment of Survivor Concerns Scale for Gynecological Cancer Survivors: A Psychometric Study in Taiwan

**DOI:** 10.1097/jnr.0000000000000323

**Published:** 2019-09-20

**Authors:** Li-Yun TSAI, Kung-Liahng WANG, Jung-Mei TSAI, Shiow-Luan TSAY

**Affiliations:** 1 PhD, RN, Associate Professor, College of Nursing and Health Sciences, Dayeh University; 2 MD, Attending Physician, MacKay Memorial Hospital, Taitung Branch; and Professor, Department of Nursing, MacKay Medicine, Nursing and Management College; 3 PhD, RN, Vice Director, Department of Nursing, MacKay Memorial Hospital; Adjunct Assistant Professor, Department of Nursing, Mackay Medical College; Mackay Junior College of Medicine, Nursing, and Management; and College of Nursing and Health Sciences, Dayeh University; 4 PhD, RN, Professor, College of Nursing and Health Sciences, Dayeh University; 5 Contributed equally as corresponding author.

**Keywords:** gynecological cancer survivors, fear of cancer recurrence, assessment of survivor concerns, quality of life, confirmatory factor analysis

## Abstract

**Background:**

Most gynecological cancer survivors outlive the acute stage, and many reach permanent survival. However, the fear of cancer recurrence (FCR) is stressful and affects quality of life.

**Purpose:**

This study was designed to validate a Chinese version of the Assessment of Survivor Concerns (ASC) questionnaire in terms of its ability to assess FCR in gynecological cancer survivors.

**Methods:**

A two-stage study procedure was employed. The first stage involved the translation of the ASC questionnaire from English into Chinese using the methods proposed by Guillemin, which include translation, back-translation, consensus meetings, and a trial of potential users. In the second stage, a pilot study was completed with 37 gynecological cancer survivors followed by a psychometric property study with 287 gynecological cancer survivors. Construct validity was determined using confirmatory factor analysis (CFA) with structural equation modeling. Convergent validity was determined using composite reliability and the average variance extracted values of the ASC model. Discriminant validity was determined by comparing the model fitness of the ASC model against the model fitness of a one-construct model. Concurrent criterion validity was assessed using the European Organization for Research and Treatment of Cancer's Quality-of-Life Questionnaire Core 30 as the auxiliary instrument. Reliability was determined by measuring the internal consistency reliability using Cronbach's α in addition to the 3-week test–retest reliability with a 95% confidence interval of the intraclass correlation coefficient.

**Results:**

The process of translation and back-translation was performed to ensure the conceptual equivalence of the Chinese version with the original ASC questionnaire. For CFA, the fit indices of the ASC model (χ^2^ = 9.87, *p* > .05; root mean square error of approximation = .03. comparative fit index = 1, nonnormed fit index = 1) indicated appropriate model fitness. For convergent validity, the composite reliability and average variance extracted values of the ASC model were satisfactory. For discriminant validity, the model fitness of the ASC model was significantly improved over the one-construct model. For concurrent criterion validity, the ASC scores correlated negatively with the scores of the global quality of life and the five functions (physical, role, cognition, emotions, and social) of the European Organization for Research and Treatment of Cancer's Quality-of-Life Questionnaire Core 30, as hypothesized. For reliability, the Cronbach's α and the 95% confidence interval of intraclass correlation coefficient for the ASC model were .91 and [.18, .68], respectively.

**Conclusions/Implications for Practice:**

The Chinese version of the ASC questionnaire is a valid and reliable instrument that is suitable for assessing FCR in gynecological cancer survivors in clinical and research settings.

## Introduction

Advances in the early detection and treatment of cancer have increased the chances of survival of patients with cancer significantly. The United States had an estimated 14 million cancer survivors in 2014, underscoring that cancer survivor care is a challenging and important issue for the healthcare profession (Lai, 2016). Cancer survivors are under long-term psychological pressure (Handschel, Naujoks, Kübler, & Krüskemper, 2012; Kim, Carver, Spillers, Love-Ghaffari, & Kaw, 2012). One of the most disturbing emotional responses and prevalent unmet psychosocial needs of cancer survivorship is fear of cancer recurrence (FCR; Simard et al., 2013).

FCR has been recently defined as referring to “fear, worry, or concern relating to the possibility that cancer will come back or progress” (Lebel et al., 2016). With an incidence rate of 22%–87%, FCR may cause functionally impaired behaviors such as escapist behavior or recurrent symptoms and may further develop into anxiety disorder, posttraumatic stress disorder, and/or depression (Hart, Latini, Cowan, Carroll, & CaPSURE Investigators, 2008; Llewellyn, Weinman, McGurk, & Humphris, 2008; Simard et al., 2013).

The Taiwan cancer registry reported 103,147 new incidences of cancer in 2014, of which 47,054 were women. Gynecologic cancers, defined as cancers infecting the uterine corpus, uterine cervix, or ovaries in the female reproductive system, are the leading category of cancers of women domestically (Health Promotion Administration, Ministry of Health and Welfare, Taiwan, ROC, 2017). As elsewhere, gynecologic cancer survivors in Taiwan struggle with FCR and fear of death as a consequence of cancer survivorship (Tsai, Wang, Liang, Tsai, & Tsay, 2017).

Patients with cancer face relatively tangible threats such as FCR. Thus, generic anxiety scales are limited in terms of effectiveness and accuracy when used to gauge emotional responses in cancer survivorship (Groenvold et al., 1999). A systematic review identified 20 FCR-related scales and found that most had not undergone comprehensive psychometric testing (Thewes et al., 2012). The Assessment of Survivor Concerns (ASC), a brief and modular questionnaire proposed by Gotay and Pagano (2007), is one of the FCR-related scales that was developed to test a theoretical hypothesis of FCR and health issues using confirmatory factor analysis (CFA) and that was tested for reliability and validity on populations of short- and long-term cancer survivors.

Multiple studies have found various factors associated with FCR, including cancer-related factors (e.g., cancer type, severity, treatment, progression, survival time) and sociodemographic factors (e.g., health, age, education; Koch, Jansen, Brenner, & Arndt, 2013; Leake, Gurrin, & Hammond, 2001; Mirabeau-Bealeet et al., 2009; Ozga et al., 2015; Wenzel et al., 2002). All of these factors were included in the framework of this study. Quality of life (QOL) in cancer survivors was also found to be associated with FCR (Kim et al., 2012; Koch et al., 2013; Taylor et al., 2012). Therefore, this study predicted that FCR would be inversely correlated with QOL in cancer survivors and used an auxiliary scale to measure QOL.

The purposes of this study were to translate the original version of the ASC scale from English into Chinese and to determine the psychometric properties of the Chinese version of ASC (ASC-C) scale. The selection of the ASC scale as the instrument to assess FCR in gynecological cancer survivors in Taiwan was based on the clinical measurements used, population, research circumstances, scoring method, cross-cultural utilization, and scale acquisition (Waltz, Strickland, & Lenz, 2010).

## Methods

### Design and Participants

A cross-sectional study was conducted from September 2013 to December 2015. Two hundred eighty-seven gynecological cancer outpatients and members of cancer survivor support groups were recruited using purposive sampling from a medical center in northern Taiwan. The selection criteria were as follows: (a) age ≥ 20 years; (b) single cancer diagnosis in the uterine cervix, uterine corpus, or ovary by a specialist physician; and (c) having completed the first episode of treatment. Exclusion criteria were as follows: (a) having a clouded consciousness or being unable to communicate, (b) having multiple types of cancer, and (c) having a psychiatric disorder.

### Ethics Statement

The study was approved by the institutional ethical review board of MacKay Memorial Hospital (13MMHIS035). Written informed consent was collected from all of the subjects. Oral and written information about the study was given to all potential participants. The information provided to prospective participants included the purpose of the study, the possibility to withdraw at any point without any effects on current or future treatment, and the assurance that data would be presented only on a group level and that all individual data would be anonymized.

### Study Procedures

The study proceeded in two stages: the translation stage and the validation stage.

#### Stage 1: translation of the Assessment of Survivor Concerns scale

Permission to translate the ASC scale into Chinese was obtained from the original author. The translation guideline (Guillemin, Bombardier, & Beaton, 1993) was followed in a similar fashion (Tsai, Wu, Yu, Li, & Buttrey, 2014). The original English version of the ASC scale was first translated into Chinese independently by two bilingual translators. A consensus version was then synthesized by a team of oncology experts. Back-translation was subsequently performed by a bilingual translator, who was a native English-speaking nurse with no prior knowledge of the ASC scale. Both the translator and back-translator were trained in or familiar with oncology nursing. The back-translated version was compared against the original ASC scale to assess conceptual equivalence, and then the translated version was reviewed by a committee of experts to resolve any discrepancies. Subsequently, gynecological cancer survivors were asked to review and fill out the translated version, and furthermore, minor revisions were made to the scale based on their opinions. The final translation version was determined after further discussion and revision by the research team.

#### Stage 2: psychometric properties

The assessment of the psychometric properties of the ASC-C scale encompassed content validity, construct validity, concurrent criterion validity, and reliability. For content validity, five experts were invited to assess the appropriateness of each item using a 4-point Likert scale, with 4 = *extremely appropriate, no revision required*; 3 = *suitable, minor revision required*; 2 = *unsuitable, and major revision required*; and 1 = *unsuitable, deletion required* (Waltz et al., 2010). Content validity index values were determined based on the results of the experts' appraisal. A pilot study was conducted on 37 gynecological cancer patients to evaluate the clarity and difficulty of each item, the scoring procedure, and the required duration to complete the questionnaire.

For construct validity, a CFA with the structural equation modeling technique was performed to assess the hypothetical structure of the ASC scale, whereas the structural equation modeling was used to determine the convergent validity and the discriminant validity of the ASC model (Hair, Black, Babin, Anderson, & Tatham, 2006; Kim et al., 2012).

For concurrent criterion validity, the European Organization for Research and Treatment of Cancer's Quality-of-Life Questionnaire Core 30 (EORTC QLQ-C30) was used as an auxiliary questionnaire to assess the correlation of the ASC scale with the QOL of cancer survivors.

For reliability, internal consistency and test–retest reliability were evaluated. For test–retest reliability, a 3-week period was designated as the retest interval because of the normal cancer surveillance schedule of patients and the regular meeting schedule of survivor support groups. The retest sample involved 37 individuals who were selected randomly from among the recruited participants.

### Measures

#### Sociodemographic information and medical status

The demographic data that were collected from the participants included age, education, marital status, and religion, whereas the collected medical status data included cancer type, stage, survival time, treatment, and severity as well as sleep quality.

#### Assessment of Survivor Concerns scale

The primary scale in this study was the ASC scale, which contains six items in the two subscales of cancer worry and health worry. In its original form, the cancer worry subscale contains the three items of “*I worry about future diagnostic tests*,” “*I worry about another type of cancer*,” and “*I worry about my cancer coming back*,” and the health worry subscale contains the three items of “*I worry about dying*,” “*I worry about my health*,” and “*I worry about my children's health*.” The cancer worry subscale focuses on FCR, whereas the health worry subscale focuses on fears related to health issues or QOL. All items are rated on a 4-point Likert scale, with 1 = *not at all*, 2 = *a little bit*, 3 = *somewhat*, and 4 = *very much* and with higher scores indicating greater worry. In the ASC-C, the sixth item, “*I worry about my children's health*,” was modified to “*I worry about my family's health*” to better reflect the culture or social situation in Taiwan. The ASC-C scale possesses the psychometric properties in reliability, with cancer worry (*α* = .93) and health worry (*α* = .63), and in construct validity, as supported by substantial correlations with auxiliary criterion measures.

#### European Organization for Research and Treatment of Cancer's Quality-of-Life Questionnaire Core 30 Taiwan Chinese version

The 30-item EORTC QLQ-C30, developed to assess health-related QOL in patients with cancer, includes the five functional subscales of physical, cognitive, social, emotional, and role; a symptom subscale; and a global QOL subscale (Fitch, Gray, & Franssen, 2000; Shinn et al., 2009; Stavraka et al., 2012; Zeng, Ching, & Loke, 2011). The score of each subscale ranges from zero to 100, with lower functional scores indicating worse global health status or worse functional status and higher symptom scores indicating worse symptom status. The Taiwan Chinese translation of EORTC QLQ-C30 exhibits good reliability, with Cronbach's α ≥ .70 for all subscales with the exception of cognitive function (Chie, Yang, Hsu, & Yang, 2004).

### Statistical Analyses

Scores were calculated based on the total ASC-C scale score and the manual of EORTC QLQ-C30 scale. SPSS Version 19 was used to conduct descriptive statistics, independent *t* test, and one-way analysis of variance. LISREL (Version 8.54) was used to estimate and test CFA.

## Results

### Demographics and Assessment of Survivor Concerns Characteristics

The characteristics of the participants and the ASC-C scores are summarized in Table 1. The largest number of participants was between 51 and 60 years old (*n* = 111, 38.95%), was educated to the junior high school level or below (*n* = 117, 41.05%), was married (*n* = 187, 65.61%), and held religious beliefs (*n* = 220, 77.19%). In terms of cancer history, the most common cancer site was the uterine cervix (*n* = 122, 42.81%), and the most common stage at diagnosis was Stage I (*n* = 182, 66.67%). Most of the cancers were of average severity (*n* = 126, 44.52%) and were treated by surgery (*n* = 119, 41.75%). Nearly half of the participants had a cancer survival duration of over 5 years (*n* = 133, 47%). Most of the participants self-reported a normal QOL (*n* = 120, 42.11%) in the realm of sleep quality.

**TABLE 1.**
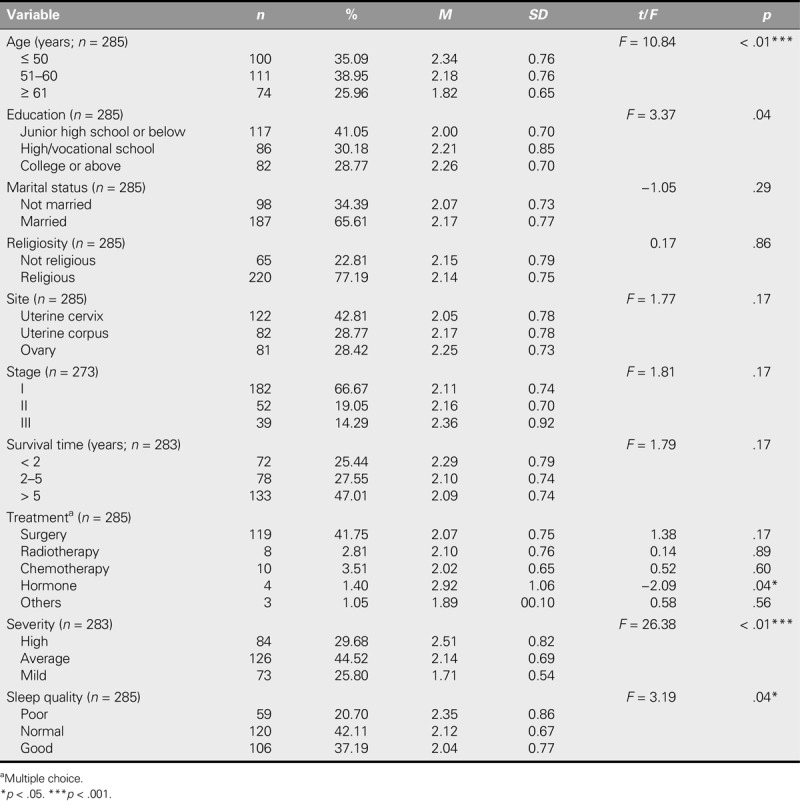
Participant Characteristics and the Chinese Assessment of Survivor Concerns Scale Scores (*N* = 287)

### Distribution of Responses on the Chinese Version of the Assessment of Survivor Concerns Scale

High percentages of the participants self-reported a low score (i.e., “a little bit” or “not at all”) for the following items: “*I worry about future diagnostic tests*” (78.74%), “*I worry about another type of cancer*” (75.26%), and “*I worry about my cancer coming back*” (72.12%) on the cancer worry subscale and “*I worry about dying*” (79.44%) and “I worry about my health” (69.34%) on the health worry subscale. Conversely, the response to the item “*I worry about my family's health*” on the health worry subscale was significantly higher, with 84.31% self-reporting as “a little bit,” “somewhat,” or “very much” worried. The detailed distribution of ASC-C scores is shown in Table 2.

**TABLE 2.**
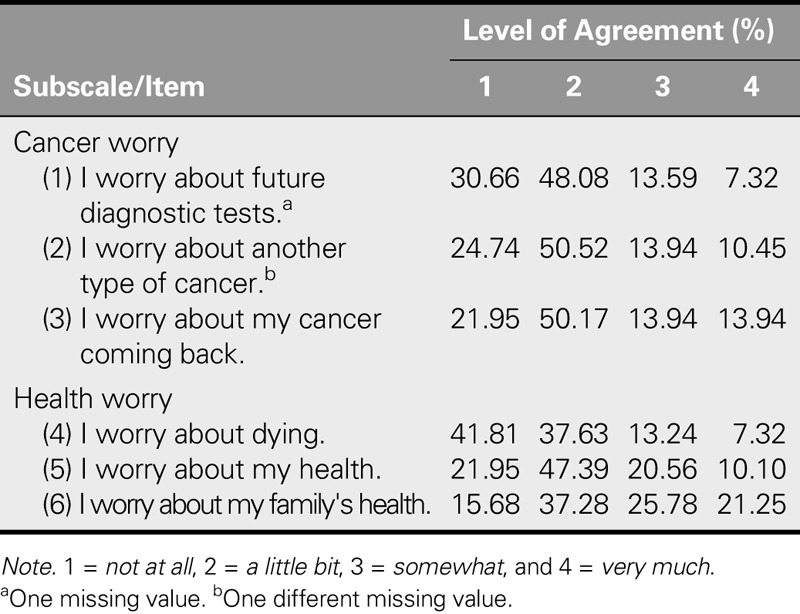
Frequency Distribution of Participants' Level of Agreement on the Chinese Assessment of Survivor Concerns Scale Items

### Validity Testing

#### Confirmatory factor analysis and construct validity

We conducted a series of CFAs to assess the goodness of fit of measurement structures and the construct validity of the ASC model with the observed data set. Construct validity and discriminant validity were evaluated using convergent validity and CFA, respectively. Convergent validity was supported by the average variance extracted (AVE) and composite reliability (CR) values of each construct, whereas discriminant validity was supported by the difference in goodness-of-fit indices between the ASC model and a one-construct model (Hair et al., 2006). Several indices were used to determine model fitness, including the chi-squared likelihood ratio statistic (χ*^2^*), comparative fit index (CFI), nonnormed fit index (NNFI; aka Tucker–Lewis index), and root mean square error of approximation (RMSEA). The cutoff criteria for assessing model goodness of fit were as follows: ≥ .90 for CFI, ≥ .95 for NNFI, and ≤ .06 for RMSEA. Furthermore, the thresholds for the satisfactory values of AVE (> .5) and CR (≥ .7) were obtained (Hair et al., 2006; Hu & Bentler, 1999).

Both the factor of cancer worry (AVE = .75, CR = .9) and of health worry (AVE = .57, CR = .79) reached the preset threshold, providing evidence for convergent validity. In addition, the fit indices of the ASC-C model (χ^2^ = 9.87, *df* = 8, *p* = .27, CFI = 1.0, NNFI = 1.0, RMSEA = .03) both met the preset criteria and were significantly better than those of the one-construct model (χ^2^ = 24.30, *df* = 9, *p* = .004, CFI = .99, NNFI = .98, RMSEA = .08). Therefore, discriminant validity was supported.

#### Concurrent criterion validity

Table 3 shows the correlation between the ASC-C scale and the Taiwan Chinese version of the EORTC QLQ-C30. The total and subscale scores of the ASC-C scale both correlated negatively with the five functions and the global QOL of the EORTC QLQ-C30 scale. Furthermore, ASC-C scale scores were correlated with the symptom scores of the EORTC QLQ-C30 scale. All of the correlations were weak, with the exception of the emotional function and global QOL scores, indicating that FCR affects the emotions and global QOL of cancer survivors.

**TABLE 3.**
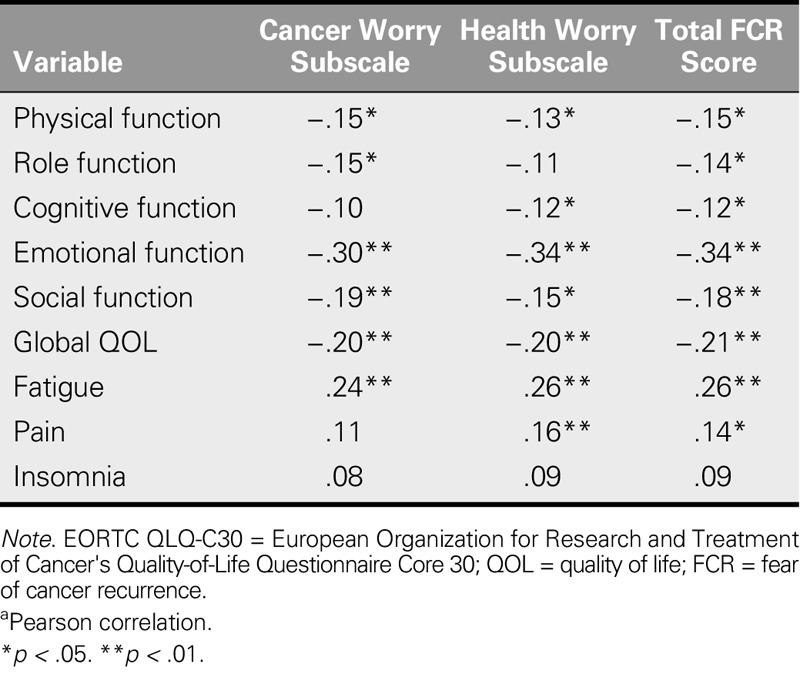
Correlation of the Chinese Assessment of Survivor Concerns and EORTC QLQ-C30 Taiwan Version^a^ (*N* = 287)

### Reliability Testing

#### Internal consistency and test–retest reliability

Table 4 shows the results of the reliability assessment of the ASC-C scale. Internal consistency was confirmed using this criterion: Cronbach's alpha > .7. Reliability was confirmed using this criterion: corrected item–total correlation value of .5–.85 (satisfactory). Test–retest reliability was confirmed using this criterion: intraclass correlation coefficient based on a mean-rating, absolute-agreement, two-way mixed-effects model with a 95% confidence interval value of .5–.75 (satisfactory).

**TABLE 4.**
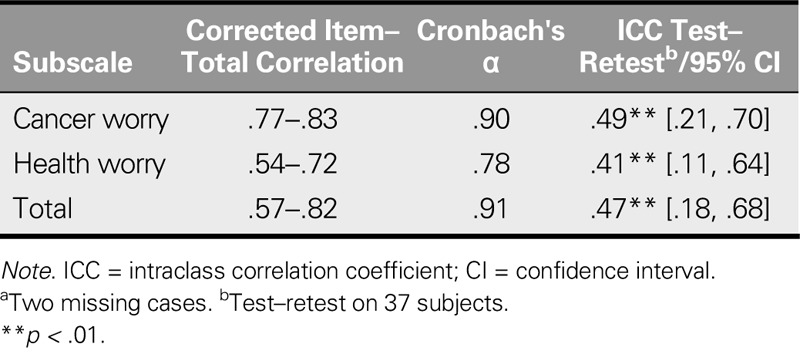
Reliability of the Chinese Assessment of Survivor Concerns Scale (*N* = 287^a^)

The good reliability of the ASC-C scale was supported by the following results: Cronbach's alpha coefficients for the cancer worry and health worry subscales and total scale scores of .90, .78, and .91, respectively; obtained corrected item–total correlation values between .54 and .83; and obtained intraclass correlation coefficient's 95% confidence intervals for cancer worry, health worry, and total scale scores of [.21, .70], [.11, .64], and [.18, .68], respectively.

## Discussion

This study completed a translation and cultural adaptation of the ASC questionnaire from its original English version into Chinese and validated the translated version using a sample of 287 gynecological cancer survivors. The psychometric properties of the ASC-C scale were supported in terms of good content validity, internal consistency, test–retest reliability, and concurrent criterion validity, whereas the construct validity of the scale was shown by convergent validity and discriminant validity through CFA. To compensate for the heavy cross-loading between the two subscales of the ASC model, the basic approach chosen for discriminant validity (comparing the goodness-of-fit indices of the ASC model and a one-factor model) was less rigorous. Hence, the cross-loading of the ASC model was also explored using the observed data set. We modified the health structure of the ASC model using the following additional constraints: (a) added correlations between the error variances of the items “*I worry about my health*” and “*I worry about dying*” and between the error variances of the items of “*I worry about my family's health*” and “*I worry about dying*” and (b) fixed the error variance of “death” to a value computed by the arithmetic formula (1 − reliability) × standard deviation. The modified model passed the strenuous validation methods for construct validity using CFA. Nevertheless, it is believed that, because of its strong, theory-based foundation, the original structure of the ASC model is highly suited to investigating the cancer recurrence concerns of cancer survivors.

The sample of gynecological cancer survivors in this study exhibited unique characteristics. The variables of age, education, disease severity, and sleep quality were found to significantly affect FCR scores. Moreover, the participants who were 50 years old or less had a higher mean score than those who were 51–60 years old, who, in turn, had a higher mean score than those who were over 60 years old, indicating that younger participants had higher levels of FCR than older participants. This finding is similar to those of earlier studies (Crist & Grunfeld, 2013; Fitch et al., 2000). The finding that survivors with an educational level of college or above had higher FCR scores also echoed the findings of previous reports (Koch et al., 2013). Cancer severity and sleep quality were found to correlate with FCR, with greater cancer severity correlated with higher levels of FCR. This finding is consistent with a positive correlation between cancer severity and FCR identified in Kim et al. (2012) and Stavraka et al. (2012). Although most gynecological cancers are diagnosed after menopause, cancer treatments frequently trigger early menopause, which may be a factor in the generally poor sleep quality reported among gynecological cancer survivors. This study similarly found that poor sleep quality was associated with greater FCR, which coincided with sleep difficulties and may aggravate psychological stress (Fitch et al., 2000). Furthermore, the large percentage (47.01%) of our participants who were long-term (≥ 5 years) cancer survivors contrasted with the original study of the ASC questionnaire (Gotay & Pagano, 2007), in which 78.6% of the participants had been cancer survivors for a shorter duration (1.5–2.5 years).

The sample of gynecological cancer survivors in this study reported a significant level of FCR. The most significant ASC items, ranked in descending order, were as follows: “*I worry about my family's health*,” “*I worry about my cancer coming back*,” “*I worry about my health*,” “*I worry about future diagnostic tests*,” “*I worry about another type of cancer*,” and “*I worry about dying*.” This list of priority concerns parallels the findings of previous studies, in which worry about family members (Zeng et al., 2011), loss of hope (Shinn et al., 2009), and stress of cancer recurrence long after cancer diagnosis (Kornblith et al., 2007; Llewellyn et al., 2008; Wenzel et al., 2002) top the worry list of cancer survivors. However, the general level of cancer worry that was observed in this study was lower and more closely clustered than that observed in the data set that was used in the original study on the ASC questionnaire (Gotay & Pagano, 2007).

Several limitations of this study are noted. First, the participants were all either gynecological cancer outpatients or members of cancer survivor support groups who were recruited from one medical center in northern Taiwan. Thus, this sample may not represent the diverse experiences of cancer survivors in the general population, and caution should be taken in interpreting or generalizing the results. Second, our examination of the QOL of gynecological cancer survivors excluded certain health-related issues such as fertility. This exclusion narrowed the breadth of this investigation. Finally, the criterion validity and sensitivity of the ASC-C scale have yet to be established.

In summary, the ASC-C scale is a brief, easy-to-use, and suitable instrument for frontline nurses to use to evaluate FCR in gynecological cancer survivors across the cancer diagnosis, treatment, and recovery stages. Using effective assessment instruments such as the ASC-C scale to screen for and determine FCR-related stress allows nurses, who are in regular contact with patients, to promptly make appropriate referrals and to provide suitable care to improve the QOL of their patients with cancer and their cancer survivor patients.
